# Integrating scanning X-ray scattering and fluorescence for multi-scale analysis of seed structure supported by machine learning tools

**DOI:** 10.1186/s13007-026-01585-8

**Published:** 2026-09-01

**Authors:** Lena Merten, Gudrun Lotze, Marianne Ahmad, Felix Roosen-Runge

**Affiliations:** 1 Division of Physical Chemistry, Lund University, Lund, Sweden; 2 Biofilms Research Center for Biointerfaces and Department of Biomedical Science, Malmö University, Malmö, Sweden; 3 Institute for Globally Distributed Open Research and Education (IGDORE), Gothenburg, Sweden; 4 Department of Oral and Maxillofacial Surgery and Oral Medicine, Malmö University, Malmö, Sweden

**Keywords:** SWAXS scanning, XRF scanning, Pea seeds, Data-driven analysis

## Abstract

**Background:**

Understanding the structure of plant seeds cultivated for human consumption and food manufacturing is vital to provide sustainable products as well as to investigate early growth stages. This includes structural variation between different plant species, varieties and cultivars depending on genetic setup, as well as structural modifications upon germination, aging and storing or seed treatment during processing. For plant seeds as multi-component biological materials, structural characterization must extend across multiple length scales, from molecular organization to cellular architecture.

**Results:**

We apply scanning Small- and Wide-Angle X-ray Scattering (SWAXS) and X-ray Fluorescence (XRF) on yellow pea seeds to combine local structural information on the molecular scale with imaging of cellular structures on the micrometer scale, enabling a comprehensive analysis of hierarchical organization. To identify and characterize heterogeneous regions within the pea seeds, we implement a fitting-free, data-driven segmentation and analysis workflow based on machine learning tools. This approach allows for classification of structurally distinct domains and enables quantitative comparison across samples without relying on predefined models. Furthermore, we incorporate multi-modal analysis by combining structural imaging with complementary elemental information obtained from XRF. The integration of compositional and structural data provides deeper insight into structure-composition relationships.

**Conclusions:**

This multi-scale, multi-modal approach opens new possibilities for investigating hierarchical structures and their development under diverse conditions and enables systematic comparison between different species or seeds at different developmental stages or exposed to different processing steps. The approach is broadly applicable to various kinds of samples and other hierarchically organized biological materials, which makes it a valuable technique for plant science as well as plant-based food science.

## Introduction

A transition to plant-based protein-rich food sources over animal products is crucial for ensuring sustainable food supply, reducing environmental strain, and addressing global hunger by providing affordable nutrition [2, 7]. Microscopic variations in seed structure among plant varieties and cultivars play an important role in identifying robust and resilient types for sustainable farming. Leguminous seeds, such as for example soy as well as yellow peas, have long been recognized as an important component of nutritionally balanced diets due to their relatively high protein content and overall nutritional value [6, 7]. Better understanding of the hierarchical structures of plant-based food sources as well as the changes and disintegration during processing is a key step in optimizing processing conditions, as well as storage environments. This extends also to vitrification processes, particularly with regard to the potential formation of ice crystals.

The cellular structure of plant seeds is specialized for nutrient storage and protection. Storage cells constitute the bulk of the seed interior, containing starch granules, which serve as the primary carbohydrate reserve [26]. Storage proteins are organized in smaller protein bodies [23, 31]. These reserves provide energy and nitrogen to support germination. The seed hull, primarily composed of structural polysaccharides such as cellulose, forms a rigid outer layer that shields the internal tissues [25, 34]. Depending on the genetic information of the plant, local variations in molecular structure and composition can be observed, which are also of considerable significance for choosing the optimal plant varieties [35].

The structures in plant seeds span multiple length scales, from the local molecular organization within the storage granules to cellular architecture. Thus, structural characterization of such materials substantially benefits from probing those length scales simultaneously in one experiment. This challenge can be addressed by scanning Small- and Wide-Angle X-ray Scattering (scanning SWAXS) with a microfocus beam, facilitating analysis of hierarchical structures and organizations [10, 21, 27, 36]. A short introduction on the structural information that can be obtained from X-ray scattering experiments can be found in the methods section. The presented experiments have been performed at a synchrotron source (ESRF, Grenoble, France, beamline ID13) during an allocated user beamtime, which is accessible to external users through a peer-reviewed proposal process.

By raster scanning the sample with a micrometer-sized beam while recording scattering patterns at each scanning point, local structural information, including molecular ordering, obtained from the scattering curve, can be combined with imaging of cellular structures. Through the resulting multi-scale structural contrast, distinct structural features on cell level and beyond can be identified and examined with respect to their molecular organization. Employing cryo-cut slices of whole pea seeds, structures and their relationships can be observed directly within the seeds in their native environment.

High resolution scanning SWAXS experiments produce big data sets. Thus, an analysis pipeline is required to facilitate fast and easy data evaluation. Traditional approaches, such as batch fitting of scattering curves, meet their limitations when structures are highly variable within one sample, can be computationally intensive, and require prior model definition. In recent years, approaches based on machine learning tools have gained popularity and have been successfully applied to scanning SWAXS data before [21]. Here, we apply such methods to the investigation of plant seed structures and create an analysis workflow which is independent of predefined models. Based on a fitting-free, data-driven segmentation, distinct regions in the sample can be identified and characterized in terms of their molecular structure.

To add an additional layer of information, the scanning SWAXS experiment is complemented by a simultaneous X-ray fluorescence (XRF) measurement. XRF can be used to determine the elemental composition of a material [15, 24]. Irradiation with high-energy X-ray photons induces electronic excitation in the atoms of the sample, which then emit secondary (fluorescent) X-ray photons at characteristic energies specific for the respective element. By spectroscopically detecting these emitted X-ray energies, the elements present in the sample and their relative amounts can be identified. Soft X-rays (lower energy) are suitable to detect light elements such as carbon, nitrogen or oxygen, whereas hard X-rays (higher energy) can be used for analyzing heavier elements. In the current experiment, hard X-rays were used, providing an optimal *q*-range for the scattering experiment. Thus, elemental information for heavier inorganic elements, such as iron, zinc, and calcium, can be obtained using XRF in this energy range.

With this approach, structural information is combined with elemental information of commonly found minerals in plant seeds to provide further insights into the co-localization of structures and certain elements. Further applications for this combination of techniques could be the observation of seeds after common processing steps involving different kinds of salts, which could be tracked in their location. This also includes processes occurring during germination and long-term storage.

**Fig. 1:**
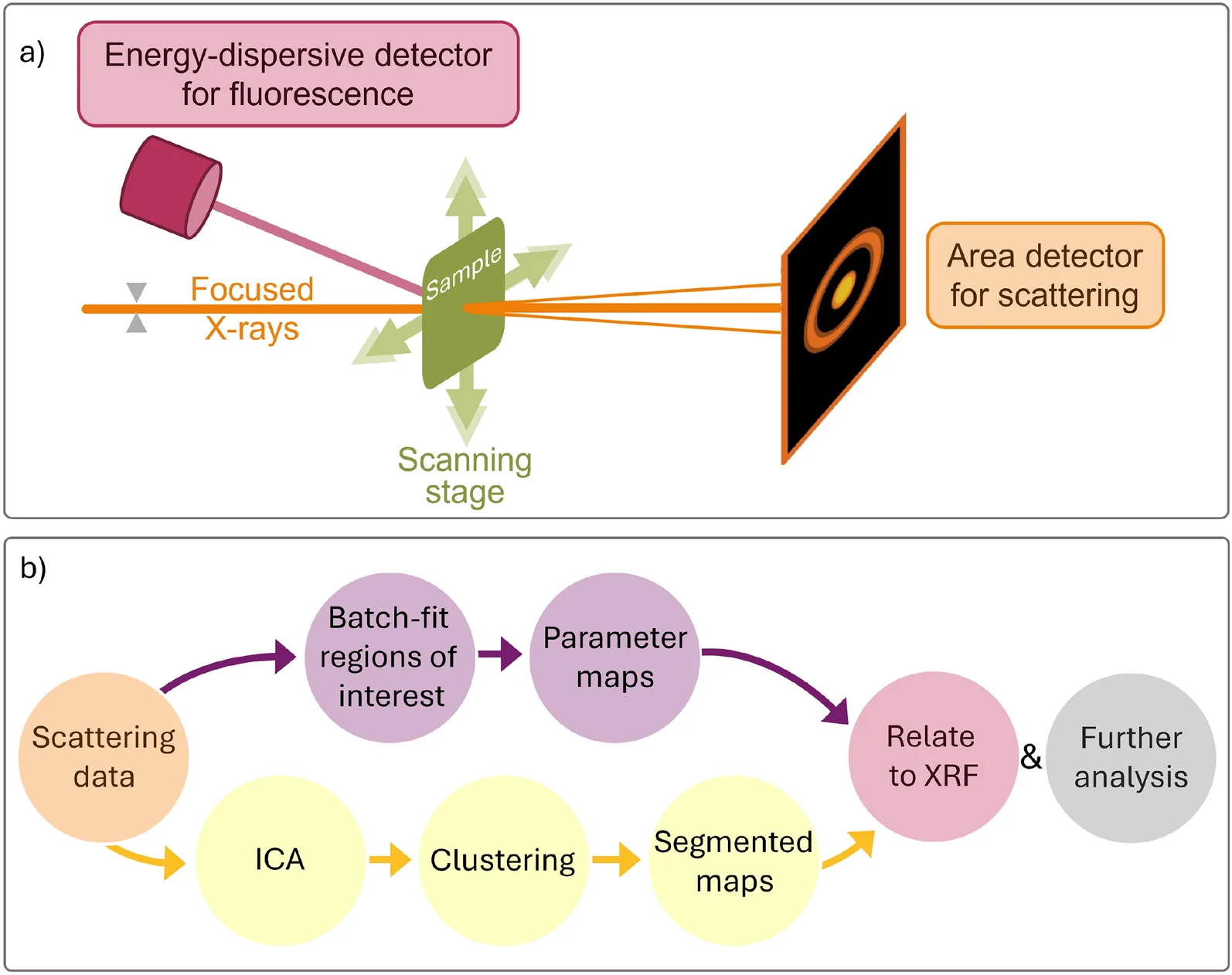
**a** Simplified scheme of the experimental configuration to simultaneously record X-ray scattering and X-ray fluorescence data using a scanning sample stage.** b** Workflow for data analysis highlighting the two different pathways based on either batch fitting the scattering curves or performing independent component analysis (ICA).

By integrating these techniques, a multi-scale, multi-modal framework is created, that is widely applicable to diverse sample types and other complex biological materials. A simplified scheme of the measuring setup is shown in Fig. 1a.

The workflow is as follows: We apply exploratory traditional data fitting to derive a preliminary estimate of structural contrast. Independently of the fitting, we perform data driven dimensionality reduction and clustering based on machine learning tools. Finally, we combine methods and datasets for a multi-modal correlative analysis and provide some perspective on additional options. A scheme of the parallel workflows is depicted in Fig. 1b.

## Materials and methods

### Sample preparation

Dry yellow pea seeds were purchased from a local supermarket (Lantmännen, brand: GoGreen, batch no. 41866). Prior to sectioning, the dried peas were soaked in water for 24 h. Each seed was subsequently bisected by a single cut perpendicular to the natural plane separating the two cotyledons, producing two equal halves. Four independent pea seeds were prepared. One half of each seed was processed for cryosectioning, while the corresponding half was processed for paraffin embedding. Multiple sections were prepared from each half-seed, and representative sections of suitable quality were selected for SWAXS analysis.

For cryosectioning, the pea seeds were mounted on a solid support plate using OCT Cryomount embedding medium (HistoLab, Sweden). The mounted samples were shock-frozen in iso-pentane without additional processing and sectioned using a Leica CM1900 UV cryotome (Leica Microsystems, USA). Cryosections of 10 *μ *m and 20 *μ *m thickness were collected on Polysine adhesion microscope slides (Germany) and subsequently transferred onto Kapton films. The thicknesses were selected to balance mechanical stability, sufficient sample volume for adequate scattering intensity, and minimal structural overlap. Consequently, the chosen thickness falls within the size range of the expected cellular structures, such as starch granules.

Paraffin embedding was used as an alternative preparation method. The corresponding pea halves were fixed in 10% neutral buffered formalin, dehydrated through a graded ethanol series, cleared in xylene, and embedded in paraffin [30]. Sections of 10 *μ *m thickness were prepared using a Leica RM2255 fully automated rotary microtome (Leica Biosystems, USA). The paraffin sections were mounted on Kapton films for beamline measurements, which served as a solid support in the beam.

A scheme of the sample preparation can be found in Figure S1, Supporting Information.

### Introduction on X-ray scattering

Various types of structural information are accessible with X-ray scattering experiments. X-rays interacting with the sample material are scattered by the electrons in the sample. In structures with a moderate degree of molecular organization, the scattered intensity can form broad features which are related to the periodicity of structural order, reflecting the average distances and correlations between structural units. Thus, the position of such broad scattering maxima reveals characteristic distances between molecules or other structural building blocks. The width of these features provides information about the degree of order: narrower peaks usually indicate stronger structural correlation and larger ordered regions. For scattering in the small-angle region, the overall shape of the scattering curve provides insight into particle size, shape, and distribution. In particular, techniques such as Small-Angle X-ray Scattering (SAXS) are useful for studying structures on the scale of nanometers, while Wide-Angle X-ray Scattering (WAXS) provides information about shorter atomic or molecular-scale distances. Overall, X-ray scattering allows to analyze materials that are partially ordered, disordered, or hierarchically structured. Comprehensive introductions for X-ray scattering techniques on biological systems can be found in literature [5, 18].

### Scanning SWAXS measurements and XRF

X-ray scattering was performed in scanning mode for several slices of yellow pea seeds. The experiments were performed at the microbranch of the beamline ID13 at the ESRF, Grenoble, France. The samples were mounted in transmission geometry, allowing for a *q*-range of *0.02-4.25* Å$^{-1}$, spanning both small- and wide-angle scattering regions. Using an X-ray energy of 13 keV, the samples were rastered in flight scans on a grid of 120 x 160 points, with a grid spacing of 2.5 *μ *m and a beam size of $2.4 \times 2.4\,\mu$m. Integration time was 20 ms, while acquisition was synchronized between the detector systems for scattering and XRF.

Scattering data was recorded using an Eiger 4M detector with a sample-to-detector distance of 163 mm.

For XRF, two VortexEM silicon drift detectors were used and combined via a XIA xMAP multi-channel analyzer system. The XRF detector system was positioned upstream of the sample at a shallow observation angle. An AZO (Aluminum doped Zinc Oxide, Al2O3:ZnO) standard was measured for normalization, while the energy ranges of interest corresponding to calcium, zinc and iron were integrated on-line (see details below).

### Data analysis

Data Analysis was performed using self-written Python scripts (version 3.12.2), employing various Python libraries and packages as detailed below.

#### Curve fitting

**Fig. 2:**
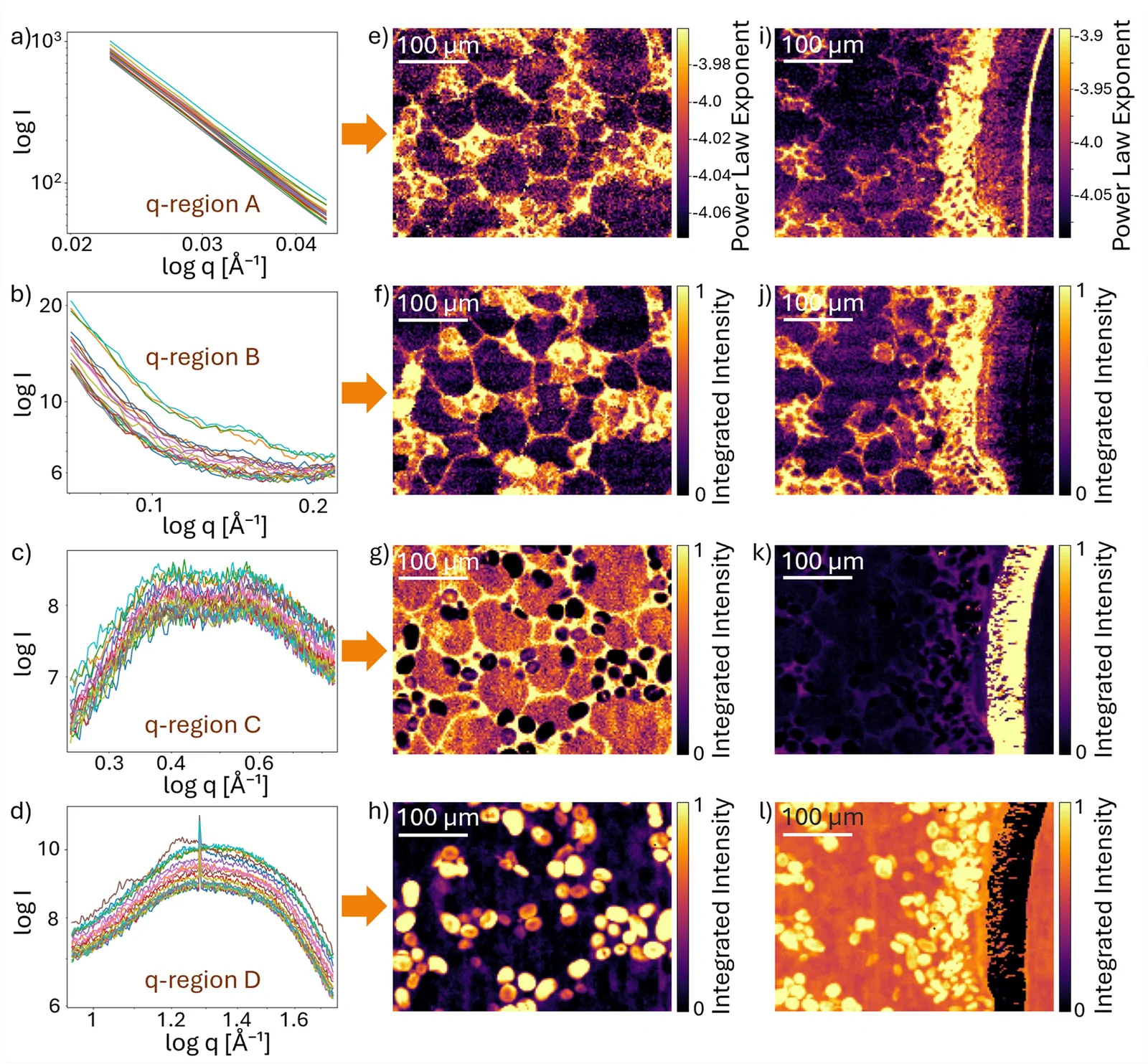
**a**–**d** Regions of interest in the data and corresponding parameter maps for a sample** e**–**h** in the interior region of the pea seed and** i**–**l** including the seed hull. The fit parameters chosen for exemplary purposes in the figure:** e**,** i** Low-*q* power law exponent,** f**,** j** shoulder integral,** g**,** k** mid-*q* peak integral,** h**,** l** high-*q* peak integral. Values were normalized to ensure consistent contrast. A selection of datapoints was chosen for the exemplary curves plotted in (**a**–**d**), which are taken at evenly spaced locations across the sample, to illustrate structural differences. More parameters and example fits can be found in Figure S2, Supporting Information, as well as the full diffraction patterns for the exemplary curves.

Before analysis, data was azimuthally integrated using the established conversion pipeline at the beamline, to receive one-dimensional scattering curves. Data fitting was performed using the *SciPy* Python library (version 1.15.1) [32], using the non-linear least squares method to fit the data to a model function within the *scipy.optimize* module. For the power law region (region A in Fig. 2), the fit function of $I(q)=\alpha *q^\beta$ was used, with fitted parameters *α * and *β *, the variable *q* representing the absolute value of the scattering vector in all equations. The shoulder area (region B in Fig. 2) was determined by numerical integration of the selected region of data, while subtracting the previously fitted power law as a background, as well as a linear flat background. The mid-*q* double peak (region C and the gray area continuing towards higher *q* in Fig. 2 and Figure S2, Supporting Information), as well as the large starch signal (region D in Fig. 2) were fitted with Gaussian functions in a multi-Gauss fit. Gauss functions were defined by $I(q) = \frac{a}{\sqrt{2\pi }\sigma } \exp (-\frac{(q-q_0)^2}{2\sigma ^2})$, with fit parameters *a*, $q_0$ and *σ *, representing the peak area, central position and width. Multiple Gaussians were combined by superposition.

#### ICA and segmentation

Independent component analysis (ICA) was performed using the *scikit-learn* Python package (version 1.6.1) [22]. The data were standardized prior to ICA, and FastICA was applied on whitened data, yielding components with approximately unit variance. The first three components were retained based on PCA-based scree plot inspection (explained variance *>95* %) and used as input for Gaussian mixture model clustering with a fixed number of clusters and full covariance. The number of clusters was selected empirically using elbow plots and silhouette analysis. Both ICA and clustering were run repeatedly to ensure stability of results. The median posterior probability of the assigned cluster label was 97.7%, while only 4.0% of points had a posterior probability below 0.6, indicating that ambiguous classifications were limited to a small fraction of the dataset, suggesting meaningful segmentation. Since the study was intended as an unsupervised exploratory analysis, the entire dataset was used for representation learning.

#### Orientational analysis

Raw detector data was used for orientational analysis and transformed to *q*-space via *PyFAI* (version 2026.2.1) [1]. Detector gaps were masked in the analysis. To calculate the degree of orientation for mapping, azimuthal profiles were exported at different *q*-values. The average deviation from the mean of the curve was used as a measure of anisotropic orientational order. The angle of preferred orientation was determined as the angle of maximum intensity after denoising the data by a rolling average (window size 11).

#### Image analysis

For object recognition, the *scikit-image* Python package (version 0.25.2) was used [33]. A binary mask was created for the desired domain for analysis based on the ICA segmentation. Object centers were identified using a Euclidean distance transform. Local maxima of the distance map were detected using a minimum separation of 5 pixels, and these maxima were used as markers for subsequent segmentation. Watershed segmentation was then applied to the inverted distance transform, using the detected markers. The watershed algorithm used default connectivity (8-connected neighborhood in 2D) as implemented in scikit-image.

#### XRF evaluation

The XRF signals in the energy regions corresponding to different elements (Ca, Zn, Fe) were integrated directly at the beamline. The XRF signal was integrated over the K$_\alpha$ fluorescence peaks of Ca (3.69 keV), Fe (6.40 keV), and Zn (8.64 keV) [13]. These elements were chosen due to their fluorescence lines in a suitable energy range, as well as their abundant presence in the sample. Data evaluation was performed using Python scripts based on *NumPy* (version 2.0.1) and *SciPy* tools.

## Results and discussion

### Curve fitting for structural contrast

To start with, a more traditional analysis of the X-ray scattering data was performed, by fitting the data with selected fit functions to receive parameters that provide information on the sample properties. Due to the large number of scattering patterns per image, batch fitting was employed using Python scripts. For the fitting, the scattering curve was segmented into four separate regions of interest, as visualized by the shaded regions in Fig. 2a–d. The regions included:

A. The low-*q* region, which was fitted with a power law function (Fig. 2a, e, i), providing information on large-scale structure and interface morphology within the sample.
B. A slightly higher low-*q* region, where a small shoulder was observed in the data. Its area was integrated after subtracting the background from the power law and an additional linear component (Fig. 2b, f, j).
C. The mid-*q* region, with a broad double peak, fitted by a double Gaussian function (Fig. 2c, g, k).
D. The higher-*q* region, which features a broad signal, that is more or less structured in different locations of the sample (Fig. 2d, h, l). It was roughly fitted as one Gaussian to be used as a background for the double Gauss fit in q-region C, and additionally fitted with a multi-Gauss fit to capture the sub-peaks in structured curves.

Figure 2 visualizes these regions, as well as chosen fit parameters mapped across two samples. Thereby, the signals from different components can be mapped to visualize their presence in the sample. The resulting maps provide a basis to co-identify locations in the maps and peaks in the scattering patterns, to ascribe the scattering features to their spatial origin. Vice versa, the regions in the map can be identified by their type of component. Two sample maps were chosen to illustrate the analysis: one in the center of a seed (Fig. 2e–h), as well as one map featuring a seed hull (Fig. 2i–l).

By considering the different fits, several domains can be identified in the map images. The larger blobs, which are for example highlighted in panel (g) by a reddish color, are recognized as the cells of the pea seed. On the contrary, the smaller elliptical domains, which appear dark in panel (g) and bright in panel (h) are identified as starch granules [26], which assemble in certain storage cells. It seems as if some storage cells in this sample might have gotten damaged during either storage or the slicing procedure, leading to free starch granules that are seemingly not associated with the cells anymore. The third major domain that can be easily identified in the images is the seed hull, which is for example prominently highlighted in panel (k).

In order to identify and characterize the structural domains in the sample, the fitted parameters in different *q*-regions were evaluated. For the low-*q* power law region (region A), the power law exponent was mapped, highlighting mainly the regions around the cells and granules, which can be observed in the images, having a lower exponent than the in-cell regions. This indicates a difference in larger-scale structures. The generally unphysical results of exponents slightly lower than *-4* are potentially related to structural anisotropy, reflecting in anisotropic scattering intensity, which is indeed present in the low-q region, as shown below in Sect. “Orientational analysis”. An influence of the background intensity cannot be ruled out, which may contribute to a general shift in the exponent values.

Region B seems to mostly (inversely) correlate with the power law exponent in region A, which could mean that the shoulder that is observed here could be related to the structures that are covering the surface of the bigger grains. A shoulder in the *q* region of *0.1-0.15* Å$^{-1}$ has been ascribed to a protein form factor before [5,] indicating that the bigger grains might be covered in small structures containing protein. Protein bodies in the size of a few micrometers have been reported to associate with the surfaces of larger starch granules, as well as found distributed within the cytoplasm [16, 29]. The signal in this region may also contain contributions from the periodic arrangement of structural cellulose or other polysaccharide fibers forming the cell walls and granule coatings [14, 18, 36, 38]. However, that would mean a similar periodicity is not observed in the seed hull, where the intensity of the shoulder is low, indicating a different kind of structure which is formed by the cellulose fibers therein.

In region C of the scattering pattern, two signals were found and fitted, which behave independently and are thus ascribed to separate structures. The lower-*q* peak of the two, which is shown in Fig. 2c, g, k, is ascribed to semi-crystalline structural polysaccharides. Those are very prevalent in the inter-cell region, building the structural framework of the tissue including cell walls, as well as in the seed hull, which is visible in panel (k) [8, 14, 17].

Lastly, the high-*q* region D is identified as relating to the starch granules, characterized by the prominent signal which is very strong and structured in the smaller granular domains, highlighted in Fig. 2h, l. An example of an instance where this signal is more structured is shown in Figure S3, Supporting Information, where the sub-peaks are identified by starch signals as found in literature [4, 11]. The size of these starch grains is approximately *20-30* *μ *m, which agrees also with those reported previously [26].

### Data-driven dimensionality reduction and decomposition

Although this method of traditional, step-wise curve fitting provides considerable insight, the curve fitting procedure of a large number of curves in batches is computationally demanding. In addition, finding appropriate starting parameters for the whole batch can be more or less complicated depending on the heterogeneity of the scattering curves, reflecting the amount of variation between the structural domains. Therefore, we investigated whether a more efficient alternative exists by applying machine learning tools to develop a fitting-free analysis pathway.

Using the Python machine learning toolkit *scikit-learn*, different techniques for dimensionality reduction, data decomposition and clustering were combined into an analysis scheme, which can identify and label different domains in the sample to investigate their properties independently.

**Fig. 3:**
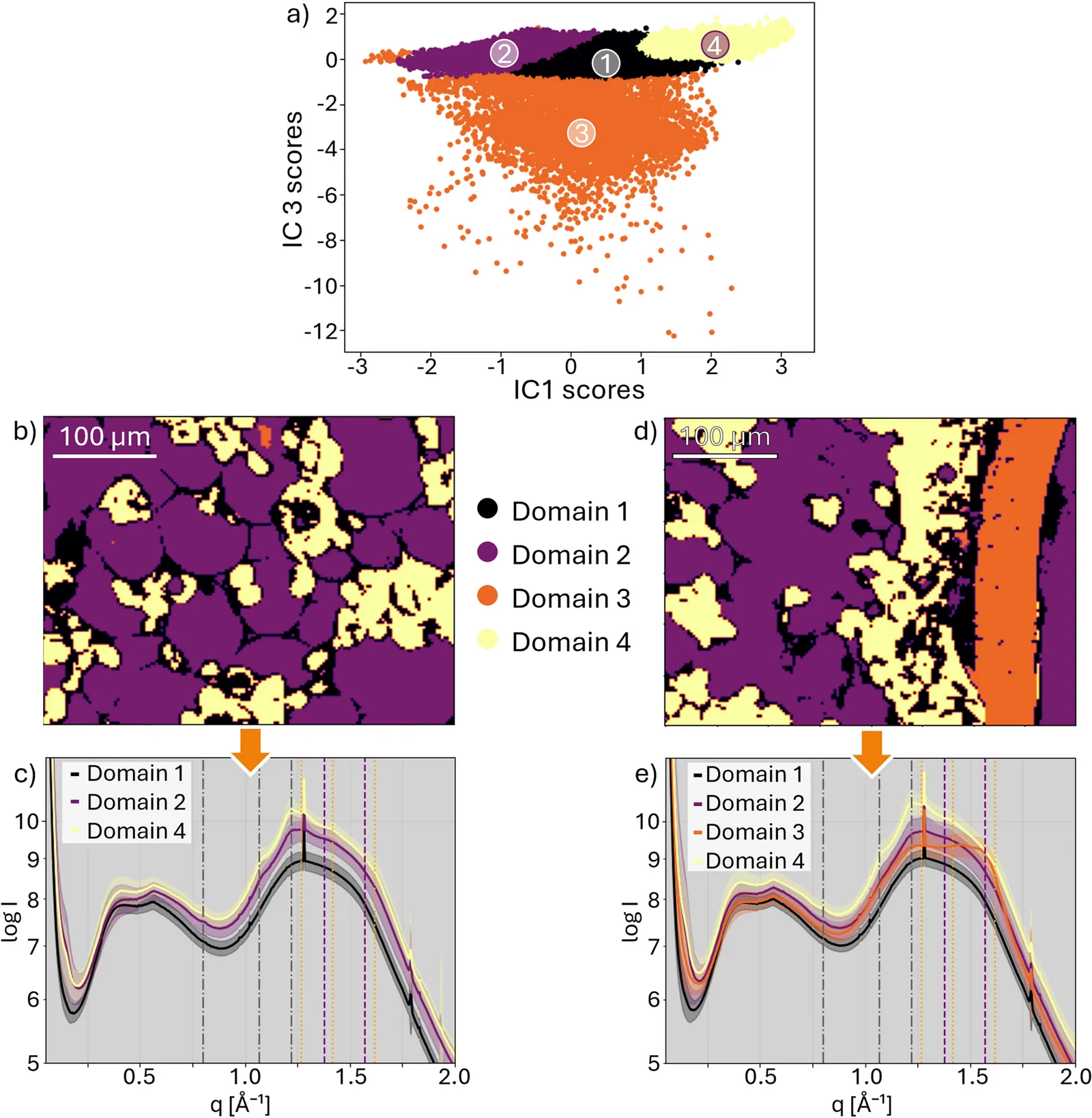
ICA-based clustering and segmentation of images.** a** Clustering of data points based on scores for independent components *1-3*, using a Gaussian mixture model.** b**,** d** Segmented sample maps based on the clustering in (**a**), for a sample (**b**) in the interior of the pea and (**d**) featuring the seed hull.** c**,** e** Characteristic (average) scattering curves for each of the segmented domains, with a shaded standard deviation interval, for the same samples as shown in (**b**,** d**). Vertical lines indicate positions of starch diffraction peaks [4, 11]. Orange, dashed: A-starch; purple, dotted: B-starch; grey, dash-dotted: shared peaks. Numbers in (**a**) correspond to the labels of regions, which were assigned arbitrarily. The maps of four samples were used in the ICA, two from the interior regions, and two edge samples. The other maps can be found in the Supporting Information, Figure S5, as well as a close-up of the low-*q* region for the characteristic scattering curves.

First, an independent component analysis (ICA) was performed on the full stack of datasets, including all datapoints from the maps of several samples. The scores of all datapoints (i.e. scattering curves) in the independent component space of the first three independent components (reconstruction error *< 5* %) were then used to cluster similar curves together and identify domains of structural similarity in the samples. Figure 3 illustrates this process. Panel (a) plots the scores of independent components (IC) 1 vs 3, which were chosen for visualization due to their favorable spread. Each data point in the plot represents one scattering curve related to one pixel of the map. The independent component curves themselves can be found in the Supporting Information, Figure S4.

A Gaussian mixture model was employed to cluster the data points into four clusters, signifying similar characteristics of the underlying scattering data. Figure 3b and d depict the sample maps colored after each pixel’s affiliation to the clusters in panel (a). Hereby we can identify the clusters with the following domains: the starch granules colored in bright yellow, the seed hull in orange, whole cells in purple, and the intercellular regions colored in black. This way we have achieved segmentation of the map into its different components, without having to fit the data to a previously chosen model or having to guess the right starting parameters. Computation time and number of assumptions made are thus drastically reduced in this approach, while a reasonable segmentation of structural domains can still be achieved.

The graphs in Fig. 3c and e show the average scattering curve of each domain in the sample, obtained by averaging of all datasets within each domain of the corresponding map. Distinct differences can be observed in the curves, characterizing structural features within each domain.

The bright yellow curves, which were collected in domain 4, identified as starch granules, show a very distinct substructure of the large peak feature between *q = 1-1.6* Å$^{-1}$. The observed peaks can be related to crystalline starch structures [4, 11], whose reported peak positions are indicated in the graph by dashed vertical lines. Different peak positions are marked for A- and B-types of starch, both of which are usually found in pea seeds, then termed as C-type starch [4, 26]. Discrimination between starch types could permit starch type imaging of inhomogeneous distributions or phase separation of starch types [3].

In contrast to the well-structured starch signal in domain 4, the purple domain 2, identified as whole cells, exhibits reduced sharpness of the peaks, while still showing starch signal. This indicates a lower amount of crystalline starch than in the starch granules, where it is supposed to be a major component. Both of these domains exhibit the low-*q* shoulder between *0.1-0.15* Å$^{-1}$, which has been ascribed to either protein signal or cellulose microfibril correlations before, agreeing with them covering the starch granules and the cells [14, 18]. A close-up of the low-*q* region for the average domain scattering can be found in the Supporting Information, Figure S5 (e).

Contrarily, in domain 1 (black), highlighting the intercellular spaces, the low-*q* signal is absent, as well as the well-ordered starch peaks. Instead, the lower-*q* part of the double peak (around *q = 0.4* Å$^{-1}$) seems to be strong in this domain, which therefore can be associated with structural polysaccharides present in the intercellular spaces and cell walls, showing a different type of structural alignment than the cellulose fibers covering the granules, probably due to an increased proportion of hemicellulose or pectin contributions [8, 17].

Finally, a significant amount of domain 3 (orange) can only be found in the sample map in panel (d), which shows the seed hull region, and therefore its characteristic curve is only shown in the corresponding plot in (e). Here it is visible that it exhibits strong signal in the region of *q = 1.5* Å$^{-1}$ to 1.6 Å$^{-1}$, which can be attributed to crystalline cellulose fibers that are prevalent in the pea seed hull [20].

In summary, these results show that the data-driven method facilitates the identification and separation of structurally distinct domains in the sample, providing insight into the dominant structural components within each domain, by directly using the scattering curves without any previous assumptions or models.

### Orientational analysis

**Fig. 4:**
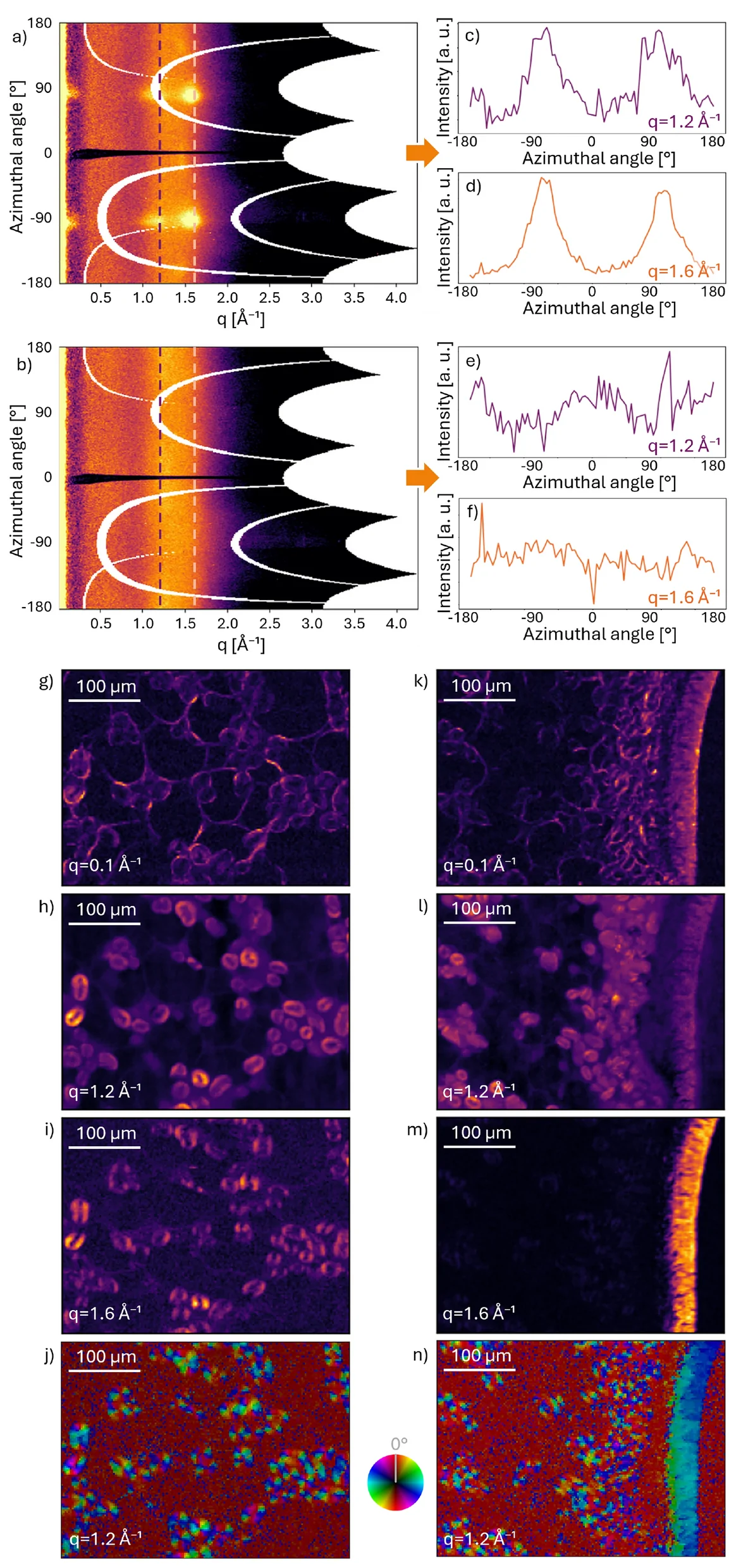
Orientational analysis. Linearized raw data from the 2D detector at a measurement spot where there is** a** strong orientational order and** b** weak orientational order. Azimuthal profiles at selected q-values of the corresponding data in (**a** and** b**):** c**,** e** at *q = 1.2 *Å$^{-1}$ and** d**,** f** at *q = 1.6 *Å$^{-1}$. Vertical dashed lines in (**a**,** b**) indicate the locations of the azimuthal profiles.** g**–**i** and** k**–**m** Maps of degree of orientational order at selected *q*-values, as indicated in the images.** g**–**i** for the interior sample,** k**–**m** for the sample featuring the seed hull.** j**,** n** Angular maximum of anisotropic intensity mapped by the colorwheel shown between the panels. The grey line indicates 0°.

So far, only the usage of one-dimensional data, azimuthally integrated from the 2D detector, has been presented. In a scattering experiment with an area detector, however, data is recorded in two dimensions, providing an additional dimension of information regarding the structural ordering and orientation of structures within the sample material. Ring-shaped diffraction features can be observed, as shown in the Supporting Information, Figure S6. A linearized projection of the same data is presented in Fig. 4a, b, with the azimuthal angle as the y-axis and the *q*-value in the x-axis. This representation enables orientational anisotropies of specific diffraction features to be readily visualized. An isotropic intensity distribution along all angles indicates randomly oriented structures, while strong azimuthal maxima in scattering intensity suggest a preferred orientation of the structure. From these azimuthal profiles, two main parameters can be extracted: the degree of orientational anisotropy and the direction of the preferred orientation.

Figure 4a and the corresponding plots in panels (c, d) are taken at a measurement spot where strong orientational order is observed, while panels (b, e, f) represent a spot with only a weak degree of orientational order for the diffraction feature at *q=1.2* Å$^{-1}$. Panels (g – l) show maps of anisotropy for different *q*-values, at which meaningful scattering signals are found. Those were obtained by taking the azimuthal intensity profiles at distinct *q*-values, as indicated in the lower left corner of each image. The average deviation from the mean intensity was used as a measure for orientational anisotropy. Anisotropy maps for more *q*-values are shown in Figure S7, Supporting Information.

From the anisotropy maps and the azimuthal analysis, we can identify three different compounds that show at least some degree of orientational order. The first is a strongly oriented compound with strongly aligned anisotropy at *q=1.2* Å$^{-1}$, *q=1.6* Å$^{-1}$ and at low *q*, (see Fig. 4a, c and d, especially highlighted in the anisotropy maps in panels (i) and (m)). This compound can be identified as cellulose, which has particularly strong anisotropy in the seed hull, mostly consisting of aligned cellulose fibers [9, 28, 37].

A second compound with orientational anisotropy is observed for the signal at *q=1.2* Å$^{-1}$ (see Fig. 4b, e, h and l), which exhibits a lower degree of order and is oriented in the perpendicular direction. This is visible in Fig. 4e by the broad maximum at around 0° as compared to the sharper maxima for the cellulose orientation along ± 90° in Fig. 4c, d. This compound seems to have observable orientational order predominantly in the outer regions of the starch granules. It could be related to some amount of ordered starch, which has a strong diffraction peak at *q=1.2* Å$^{-1}$, indicating that the outer regions of the starch granules indeed have some degree of orientational alignment. Considerable anisotropy at this *q*-value also in the hull is related to overlap with the anisotropic cellulose peak.

Finally, for the third oriented compound, a small degree of anisotropy can be detected at low *q*-values, which is especially increased in the intercellular regions (see Fig. 4g, k), and the outer edge of the seed hull. This might be related to structural polysaccharides which can be found in the cell walls between cells, forming fibrous structures that are aligned respectively to each other [28, 37].

Angular directions of spots with orientational order were determined by the maximum of the denoised azimuthal profile. As a representation, the angular distribution of the anisotropic signal at *q=1.2* Å$^{-1}$ is shown in Fig. 4j, n. It contains both contributions from cellulose, dominating in the hull, as well as contributions from starch, dominating in the starch granules. Angular distributions for other *q*-values can be found in Figure S8, Supporting Information. It can be observed that the crystalline cellulose fibers within the seed hull are all aligned respectively to each other and show a uniform angular orientation (Fig. 4j). For the starch granules, on the other hand, the angular orientation seems to be distributed around the grain, in such a way that structures are aligned radially respective to the center of the grain (Fig. 4n), potentially indicating a preferred growth direction.

Overall, using the 2D data in combination with the integrated radial scattering curves, provides another type of contrast, namely between structures with different orientational anisotropies, adding another layer of information.

### Multi-modal correlative analysis

**Fig. 5:**
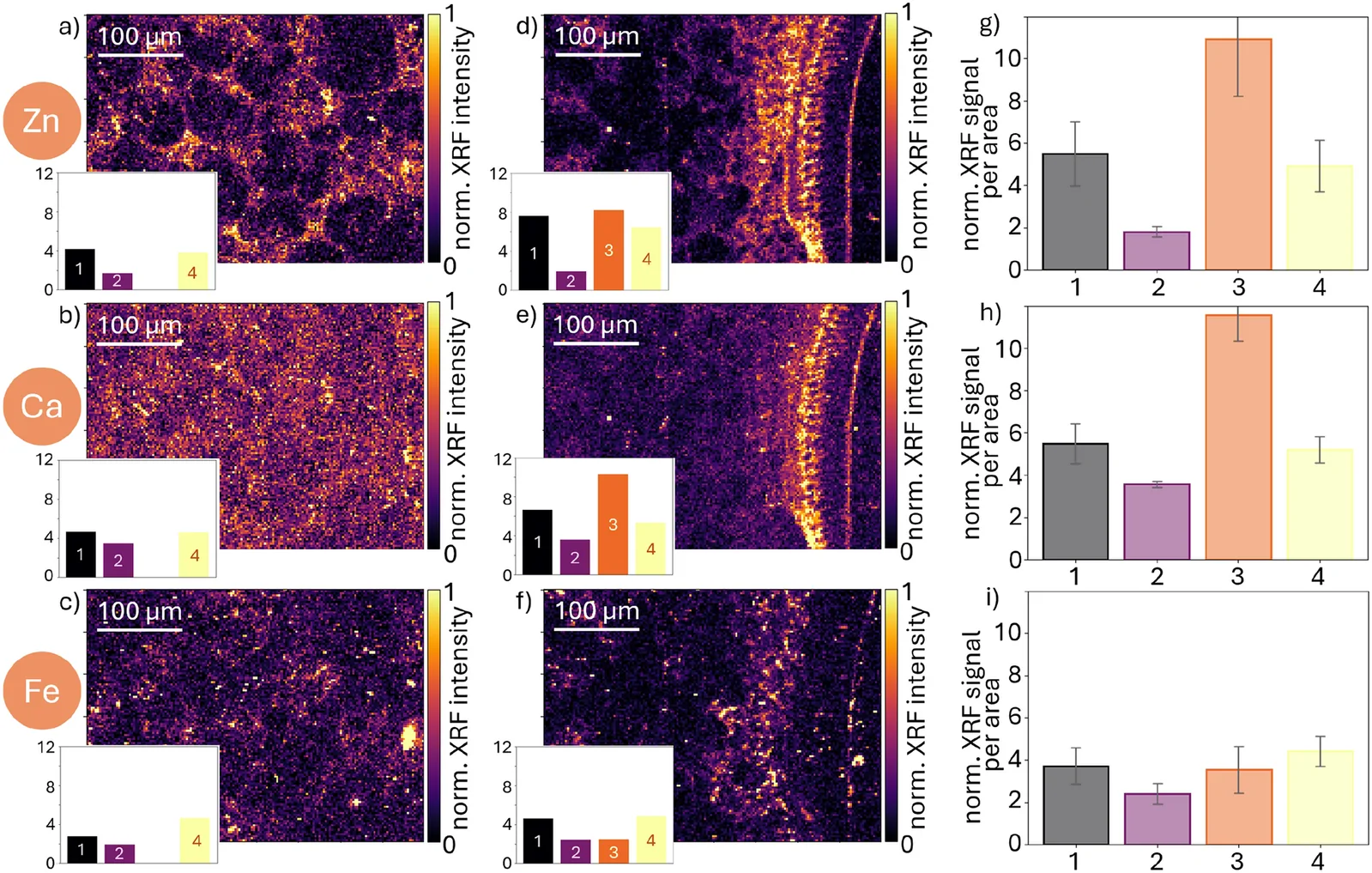
X-ray fluorescence analysis for three commonly found elements. XRF maps for (**a**,** d**) zinc, (**b**,** e**) calcium and (**c**,** f**) iron. The first column (**a**–**c**) shows the sample region on the interior of the pea while the second column (**d**–**f**) shows the seed hull region. The right column (**g**–**i**) shows the average signal per map area for the segmented structural domains as shown in Fig. 3, averaged over four samples. In samples where one region was only present in minor amounts, the corresponding region was skipped for that sample in the analysis. Averaging was performed over the map areas from four different samples, two interior and two edge samples. The insets in (**a**–**f**) show the average signals per area for the segmented domains for the specific map area. Inset axes are the same as in (**g**–**i**). The remaining XRF maps for the other samples can be found in the Supporting Information, Figure S9, while an overlay between the segmented maps and the corresponding XRF intensities is in Figure S10.

To complement the scattering data, X-ray fluorescence (XRF) was recorded simultaneously on the same samples to obtain information on the localization of specific elements within the seed. The resulting elemental maps can be related to the maps obtained from the scattering data analysis in a multi-modal analysis approach. The XRF signals of three different elements were used, namely zinc, calcium and iron, which are all commonly found in plant seeds and which are of physiological and nutritional relevance to humans [12]. Their localization in the maps of the same samples as in the other figures are shown in Fig. 5a–c for the interior area as well as for the seed hull in Fig. 5d–f. Distinct clustering and accumulation of intensity can be observed, directly related to the local prevalence of the specific element.

Figure 5g–i shows the average intensity of each element by structural domain, normalized by area. These plots have been averaged over four different sample maps, two with seed hull and two without. The small insets in Figures (a – f) show the same measure of average intensity per domain for only that specific map area. Domain colors and labels are the same as in Fig. 3. For samples where one domain was only present in negligible amounts, that domain was omitted in the analysis.

Distinct trends can be observed in the data, the most prominent of which is the accumulation of zinc and calcium on the lower part of the seed hull, which can be seen from the maps as well as the bar charts. Zinc also seems to accumulate in the intercellular regions (black domain), but less in the interior of the cells, whereas calcium seems to be more evenly distributed across the interior of the seed.

Iron shows a lower tendency to accumulate in specific regions, however a certain clustering of intensity can still be observed, indicating a preference to be located in the intercellular regions and starch granules, especially those below the seed hull, but less in the hull itself. Thus, using the fitting-free segmentation from Sect. “Data-driven dimensionality reduction and decomposition”, the localizations of the different elements can be associated with the different structural domains to identify the regions of increased prevalence.

Aside from correlating the localization to the segmented regions, a correlation analysis can be performed using the XRF intensities and the fitted parameters as obtained according to Sect. “Curve fitting for structural contrast”. The corresponding correlation matrix can be found in Figure S11, Supporting Information. This type of correlation analysis can help to find co-localizations of different elements or between elements and certain structural features.

In the current example, some correlations between parameters show clear trends, whereas others show little or no correlation. Small differences can be observed between hull and interior sample, but overall, trends are similar. Calcium and Zinc seem to be co-located, especially in vicinity of the hull region, while they both also show a weak correlation with the shoulder area and the power law exponent, indicating that they might not only be associated with the seed hull, but also with the intercellular matrix. Especially strong anticorrelation can be observed for the peak around *q=0.4* Å$^{-1}$ and the main starch signal consistently in all sample regions, clearly separating the structural scaffold of the tissue from the storage regions.

**Fig. 6:**
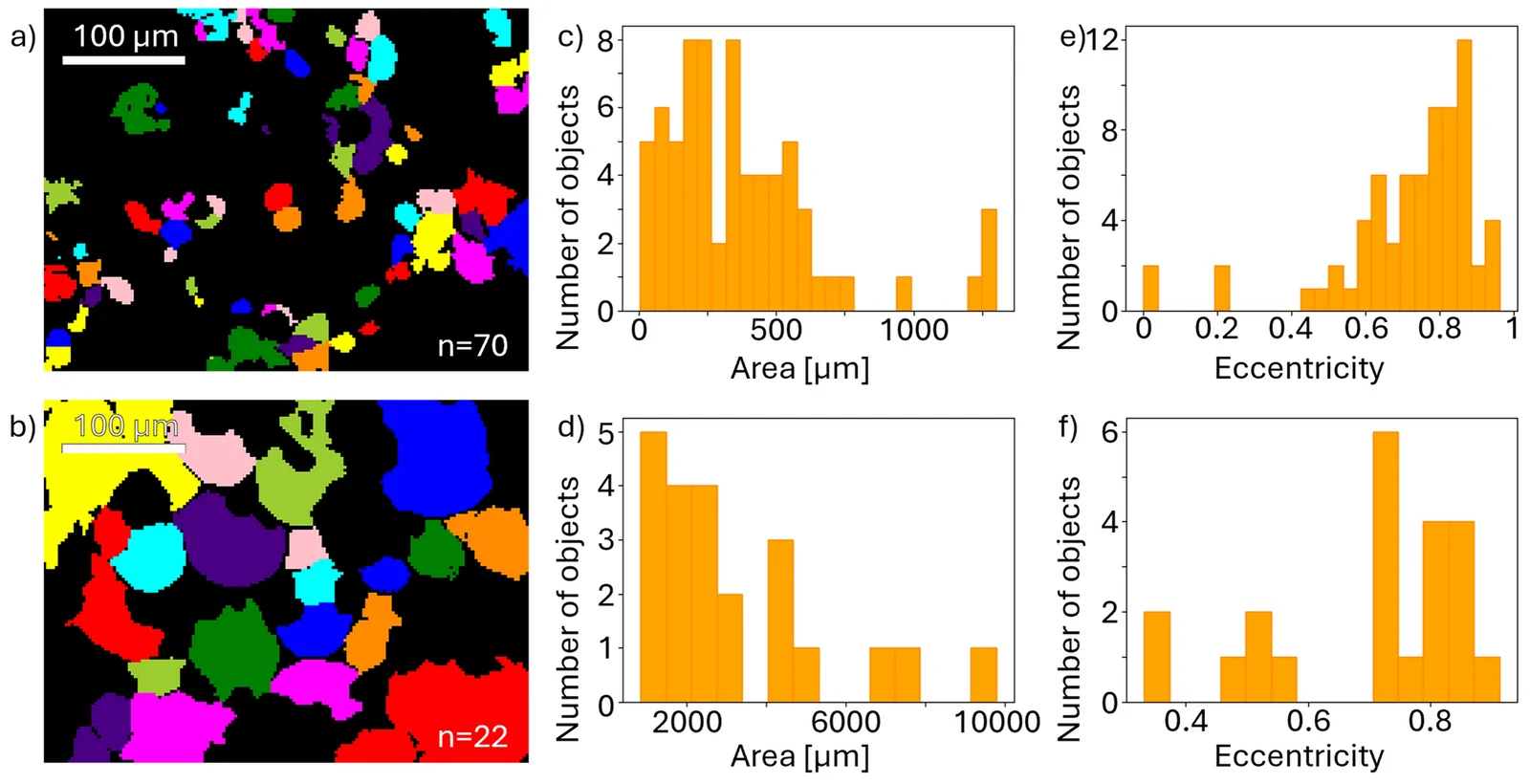
Illustration of object separation for further analysis.** a**,** b** Separated objects based on the domain segmentation from ICA, for the same sample region on the interior of the pea.** a** Separate starch granules,** b** separate cells. *n* indicates the number of objects identified in each image.** c**,** d** Histograms of size distributions of objects:** c** starch granules,** d** cells.** e**,** f** Histograms of eccentricity distributions of objects:** e** starch granules,** f** cells.

### Further analysis and outlook

Following the segmentation approach described in Sect. “Data-driven dimensionality reduction and decomposition”, the resulting domains can now serve as a basis for further data analysis. We illustrate a possible route using one of our data sets as an example for what can be done in future experiments. One way is for example to apply a simple watershed algorithm to separate the different granules or cells from each other to receive an ensemble of objects on which further statistical analyses can be performed. The resulting characteristics include parameters for shape, size, orientation and many more. A simple separation of the starch granules is shown in Fig. 6a, while cells are represented in Fig. 6b, where each identified granular object is drawn in a different color.

These segmented objects can then be used to provide information on the size and shape distributions of granules in different samples, their localizations and clustering, as well as other properties of interest. In the present case, 2D projections of the objects are obtained from the two-dimensional data. 3D information could be obtained by inverse modeling. Figure 6c - f shows histograms of the size and shape distributions of the starch granules and cells within the 2D cut. The projected area was chosen to represent the size distribution, while the shape is represented by the eccentricity of the elliptical projection of the identified objects. The number of objects (*n*), as indicated in Fig. 6a, b is relatively low, thus drawing conclusions from the current dataset is not statistically justifiable. A larger dataset would enable more meaningful conclusions.

Applicable science cases include, but are not limited to, the structural comparison of different plant species and varieties depending on genetic setup, as well as observation of structural modifications upon aging and storing, germination or seed treatment during food processing [19].

In combination with elemental analysis as presented here with XRF, also vitrification and processing steps including the distribution of salts can be elucidated, as well as a quantification of elemental composition in different tissue types, which is relevant for food safety.

Furthermore, using a nano-focused X-ray beam instead of the microfocus adopted here, resolution can be increased to resolve smaller intracellular structures such as protein bodies and other cellular components, though that might pose new challenges related to more concentrated energy deposition by the nano-beam.

While synchrotron-based imaging provides high quality data with high spatial resolution, fast acquisition and good signal-to-noise ratio, the data analysis workflow described in this study is general and can be applied to equivalent imaging data acquired using other high-resolution imaging modalities.

In terms of sample preparation, several different methods to fabricate the pea slices were tested, including cryo-cutting as well as embedding in paraffin to obtain slices of different thickness. In general, thinner slices (10 *μ *m) provided more defined data. The cryo-cut samples, which were presented in this manuscript, were preferred over the paraffin-embedded samples due to the signals from the paraffin affecting data analysis.

## Conclusions

In this study, we demonstrated that distinct structural domains within pea seeds can be effectively identified by microfocus scanning X-ray scattering, both through traditional fitting parameters and a data-driven, fitting-free approach. Machine learning-based methods proved valuable in segmenting the sample maps, enabling structural investigation of the different domains. This segmentation further facilitates quantitative analyses, such as object detection and characterization, providing a versatile tool for feature extraction within complex datasets. Moreover, combining these approaches with complementary X-ray fluorescence data offers a multi-modal framework, allowing for the co-localization and correlation of structural and compositional information. Together, this study highlights the potential of integrating machine learning techniques with conventional and multi-modal approaches to enhance spatially resolved analyses of heterogeneous and hierarchically organized samples. Importantly, performing this analysis directly on the plant seed with minimal sample processing during preparation enables the observation of structural organization and correlations of components in their native environment, as well as at different developmental stages and after processing.

## Additional file


Supplementary file 1.


## Data Availability

Data is available from the authors upon reasonable request.
