# Supplementary material for: Integrating scanning X-ray scattering and fluorescence for multi-scale analysis of seed structure supported by machine learning tools
Source: Plant Methods. 2026 Sep 1;22:78. doi: 10.1186/s13007-026-01585-8 (PMC13536852; doi:10.1186/s13007-026-01585-8)
Supplement: Supplementary file 1 [file 13007_2026_1585_MOESM1_ESM.pdf]

Supporting Information for:

Integrating Scanning X-Ray Scattering and Fluorescence for Multi-Scale Analysis of Seed Structure Supported by Machine Learning Tools

Lena Merten,[1] Gudrun Lotze,[2,3] Marianne Ahmad,[4] and Felix Roosen-Runge[1,2]

[1)] *Division of Physical Chemistry, Lund University, Lund, Sweden*

[2)] *Biofilms Research Center for Biointerfaces and Department of Biomedical Science, Malmö University, Malmö, Sweden*

[3)] *Institute for Globally Distributed Open Research and Education (IGDORE)*

[4)] *Department of Oral and Maxillofacial Surgery and Oral Medicine, Malmö University, Malmö, Sweden*

(Dated: July 14, 2026)

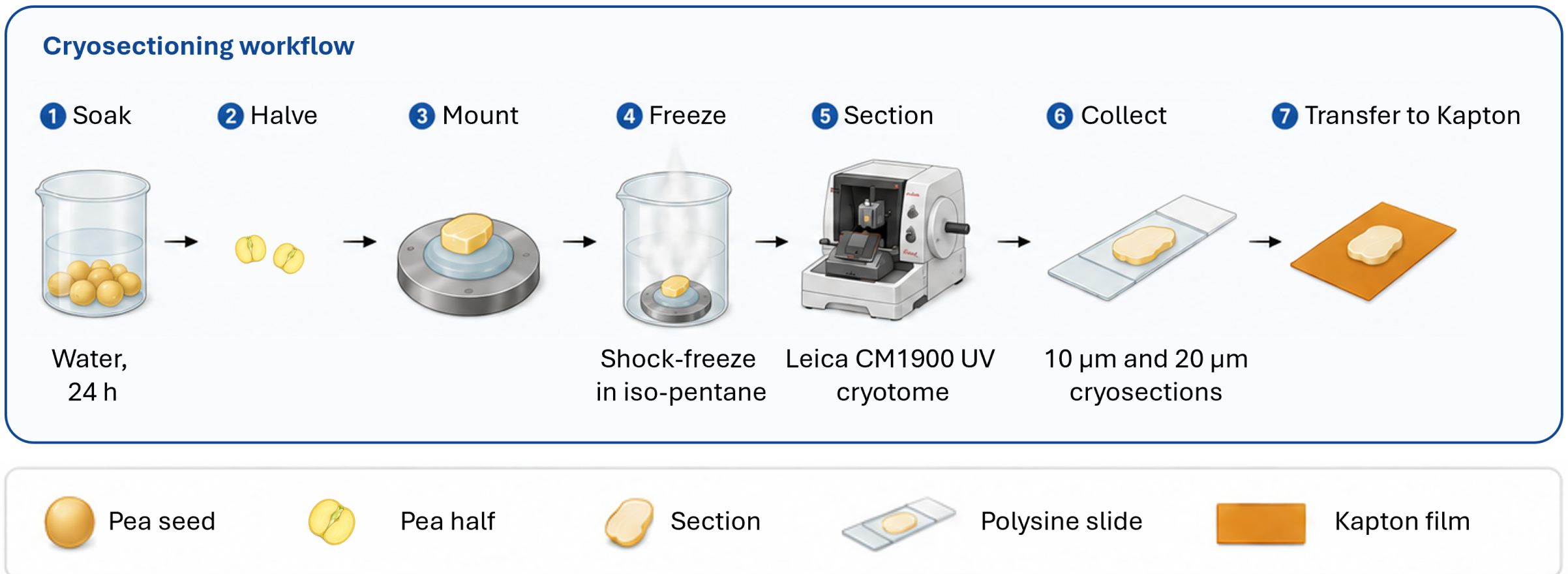

Figure S 1. Schematic depiction of the sample preparation process. (Image AI modified)

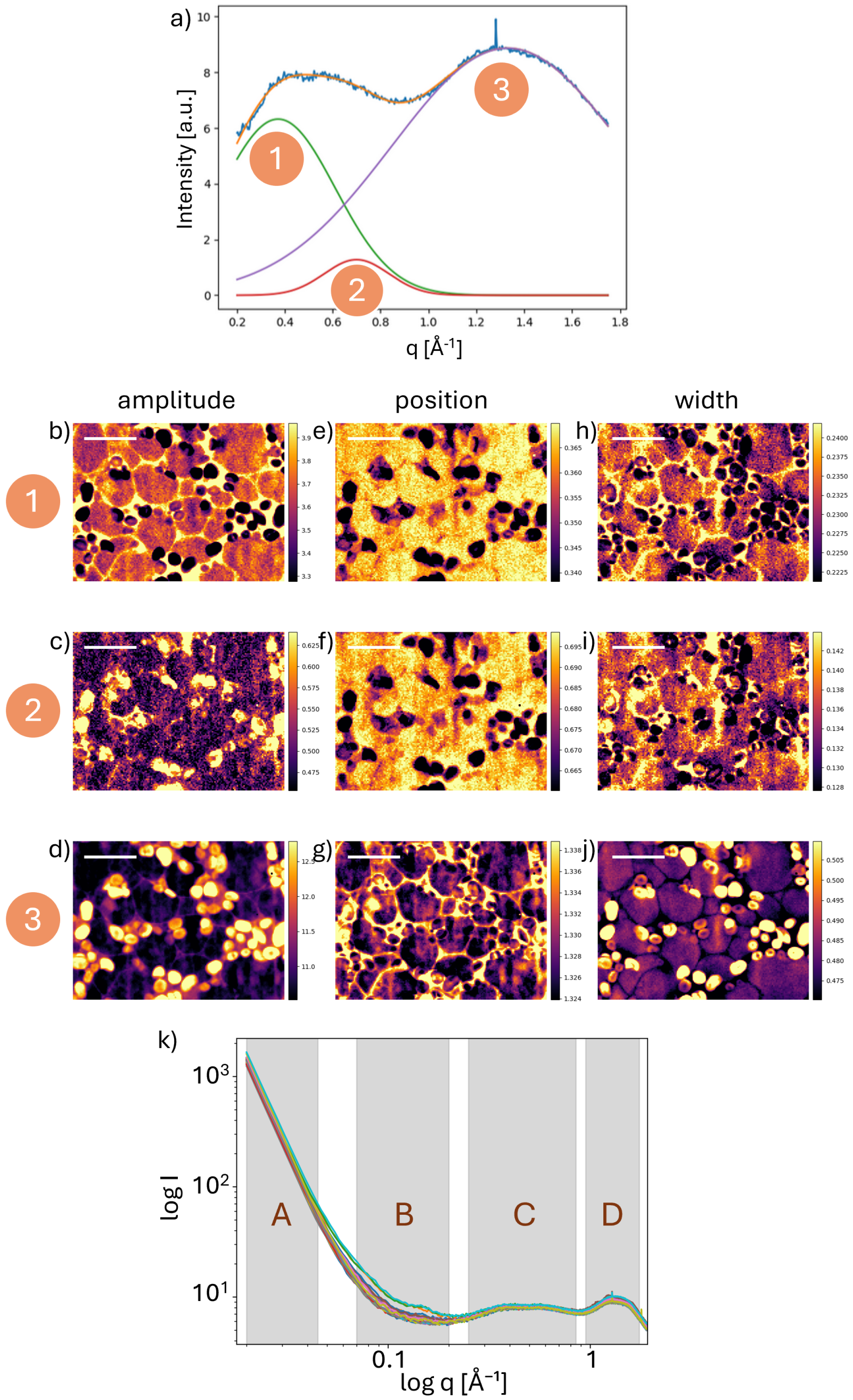

Figure S 2. (a) Example for a fit of the mid and high q region, where the large peak at high q is used as a background for the double Gauss fit of the mid q double peak. (b – j) Visualization of parameter maps from the peak fitting. White scale bars are 100 μm. (k) Full diffraction curves for the sections shown in Figure 2 (a – d) in the main text, with the sections labeled by A – D. The same selection of datapoints was chosen as for the sections in Figure 2 (a – d), which are taken at evenly spaced locations across the sample to illustrate structural differences.

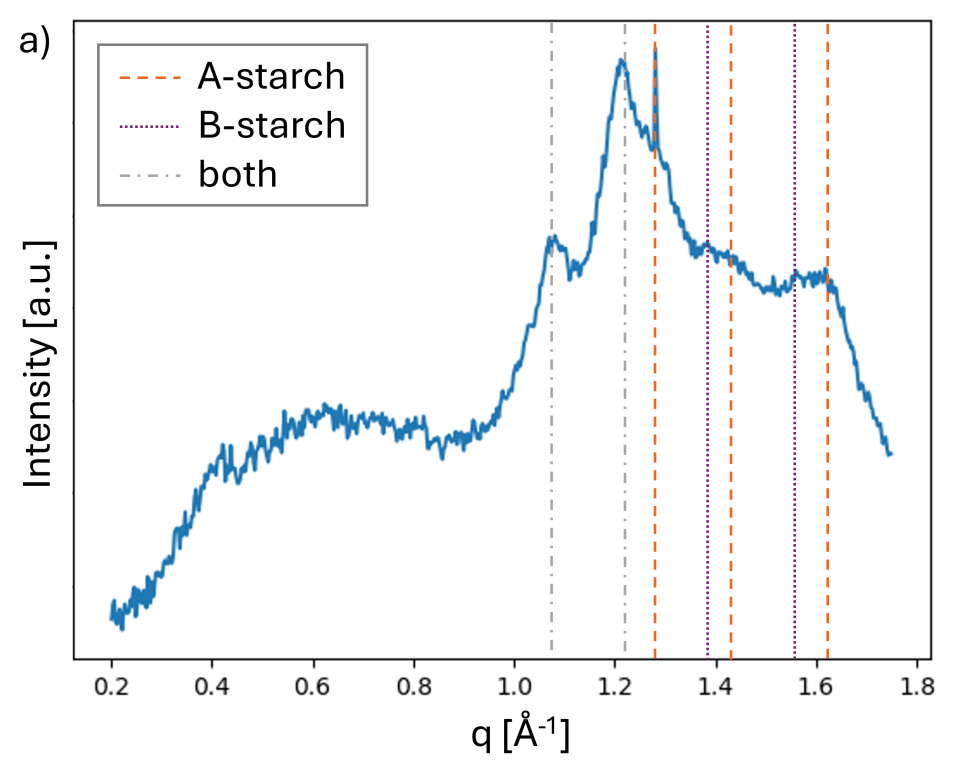

Figure S 3. Example of a scattering curve at a location where the starch signal shows exceptionally strong substructure with sharp peaks, indicating highly ordered starch in that location. Vertical lines indicate starch diffraction peaks as reported in literature.[1,2] Orange, dashed: A-starch; purple, dotted: B-starch; grey, dash-dotted: shared peaks.

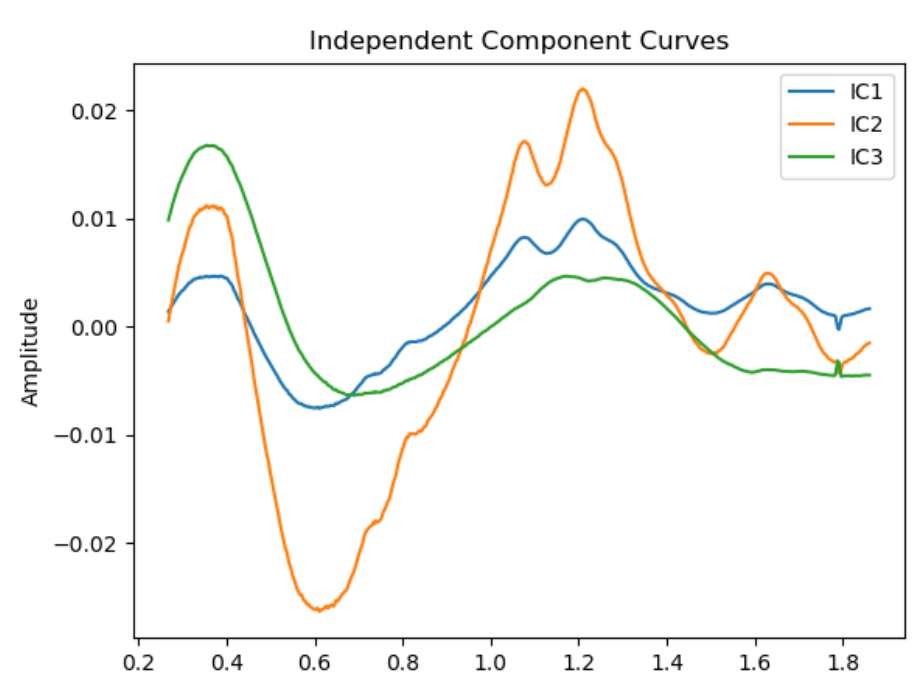

Figure S 4. Independent component curves after performing ICA on the scattering data of four samples (maps).

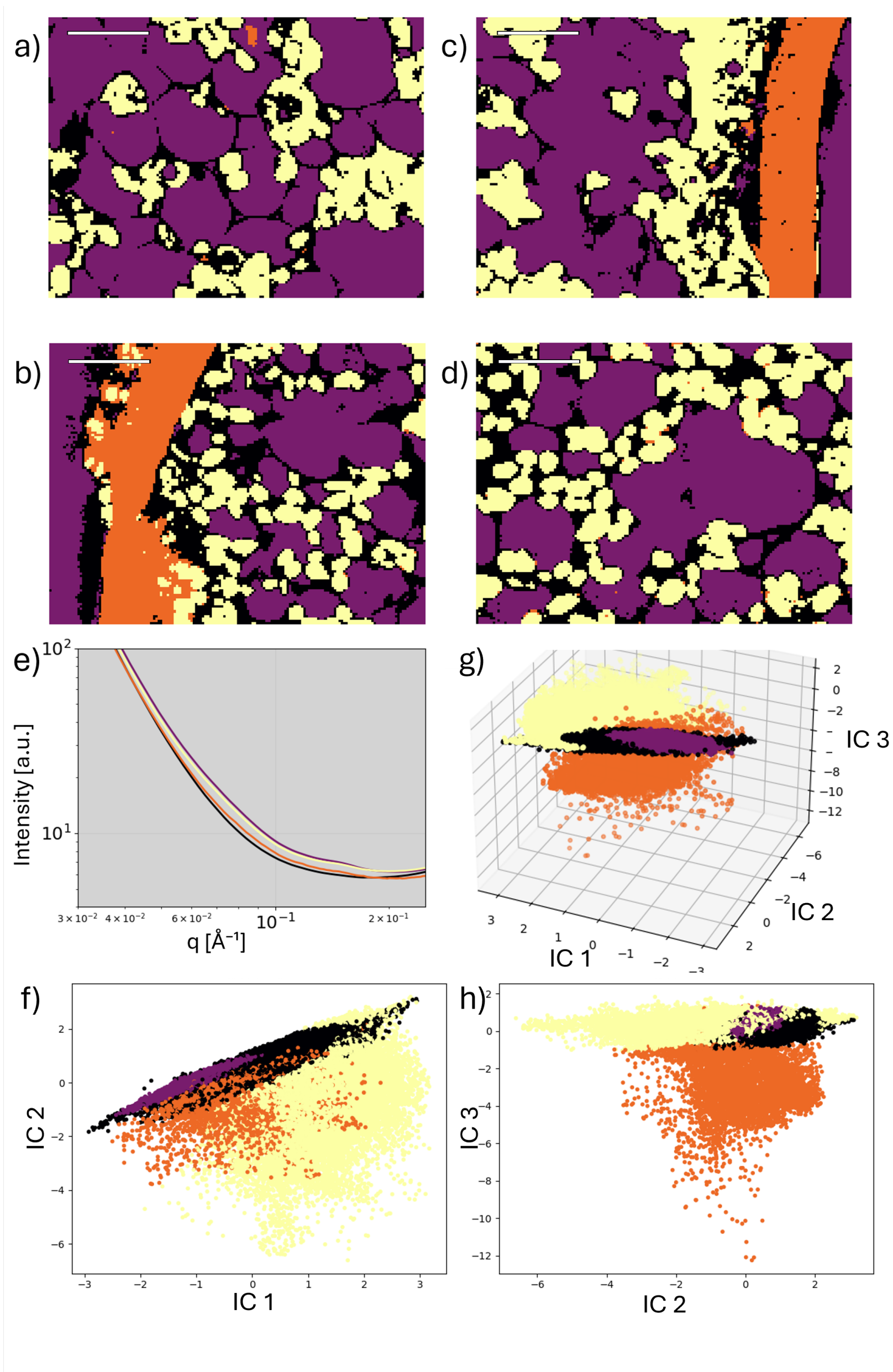

Figure S 5. (a – d) Sample maps segmented based on ICA and GMM clustering as in Figure 3 in the main text for more samples. Scale bars are 100 μm. (e) Close-up of the low-q region of the characteristic curves in Figure 3 (e) in the main text. (f, h) Projection of the clustering in (f) IC1 vs IC2 and (h) in IC2 vs IC3 dimensions. (g) 3D representation of the clustering in all three applied dimensions (IC1, IC2 and IC3)

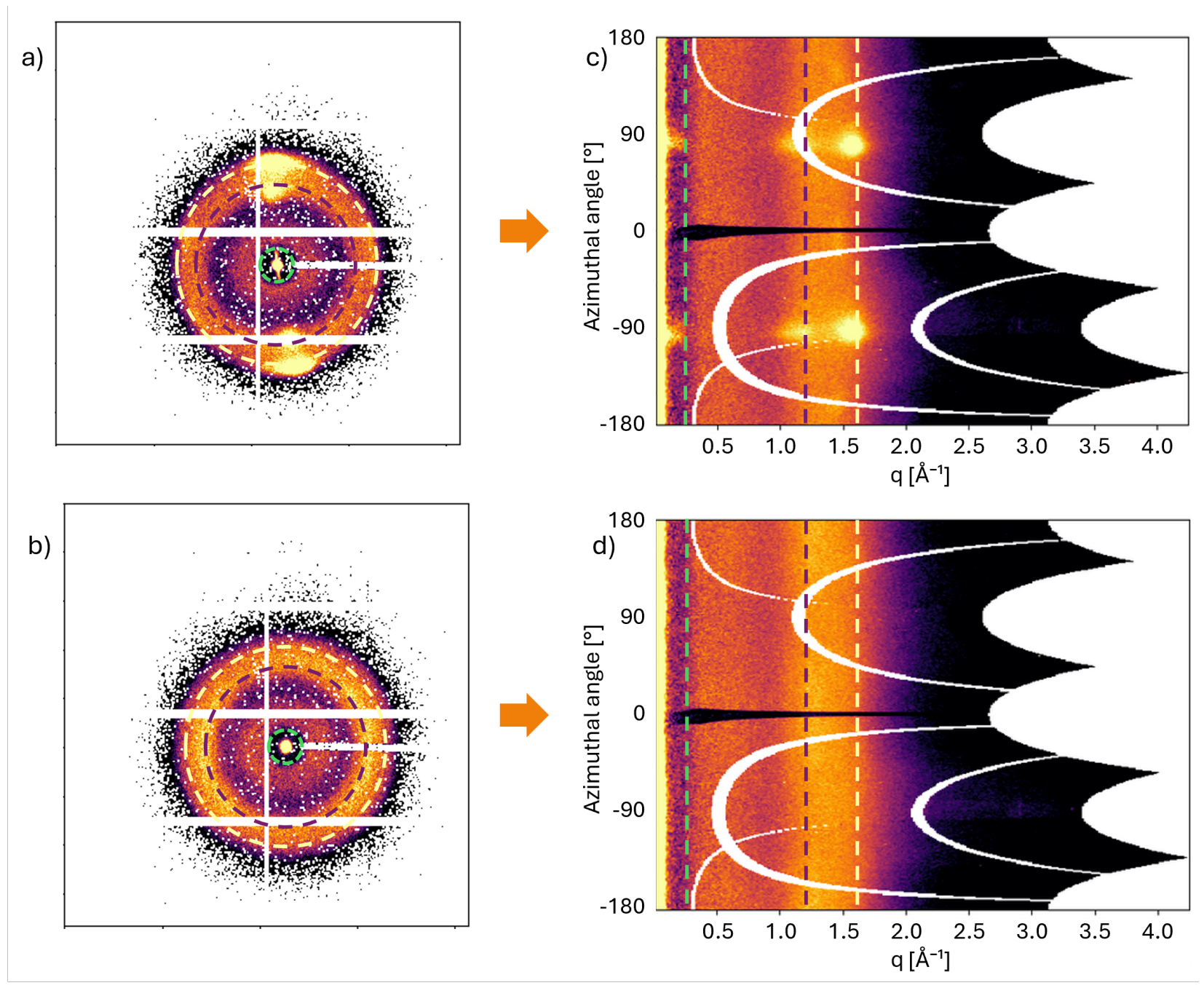

Figure S 6. (a, b) Example for raw data as received from the area detector, which is transformed to (c, d) linear maps of intensity displayed versus the azimuthal angle and q-value. Colored dashed circles in (a, b) correspond to the colored dashed lines in (c, d). Data transformation was done using PyFAI.

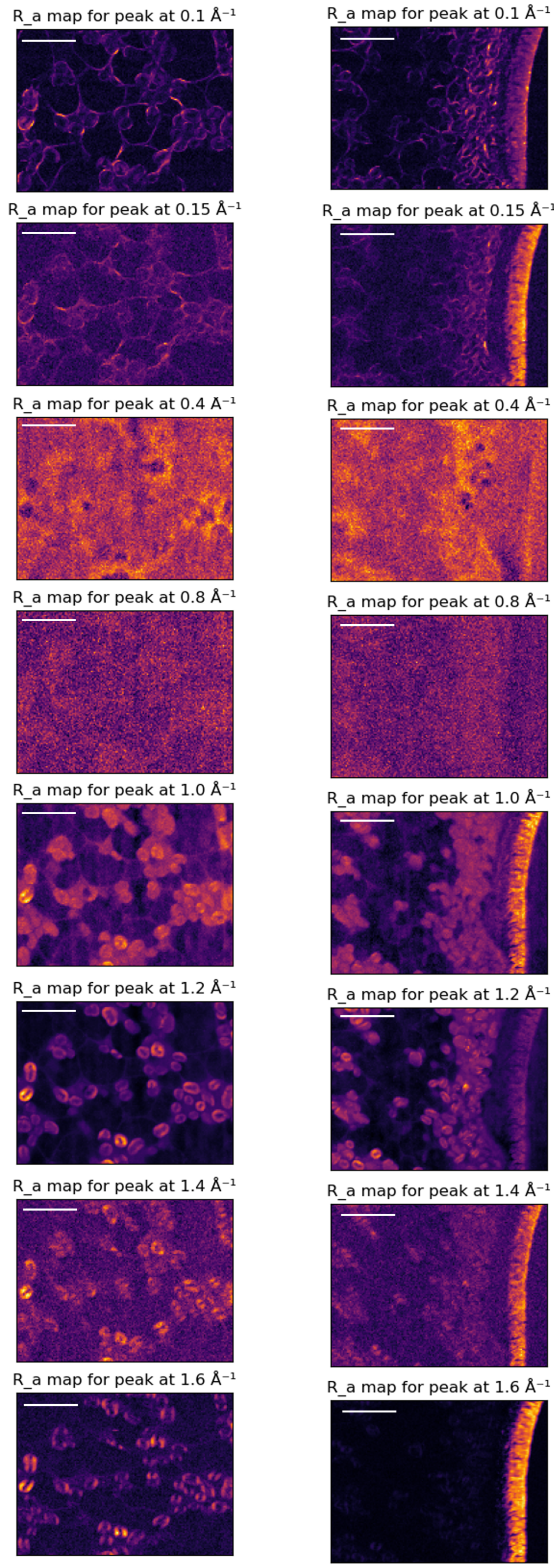

Figure S 7. Orientational anisotropy maps based on the azimuthal profiles at various q-values. The average deviation from the mean intensity of the azimuthal profile was used as a measure of orientational anisotropy. The q-values were chosen based on the observed features in the radial curves. Scale bars are 100 μm.

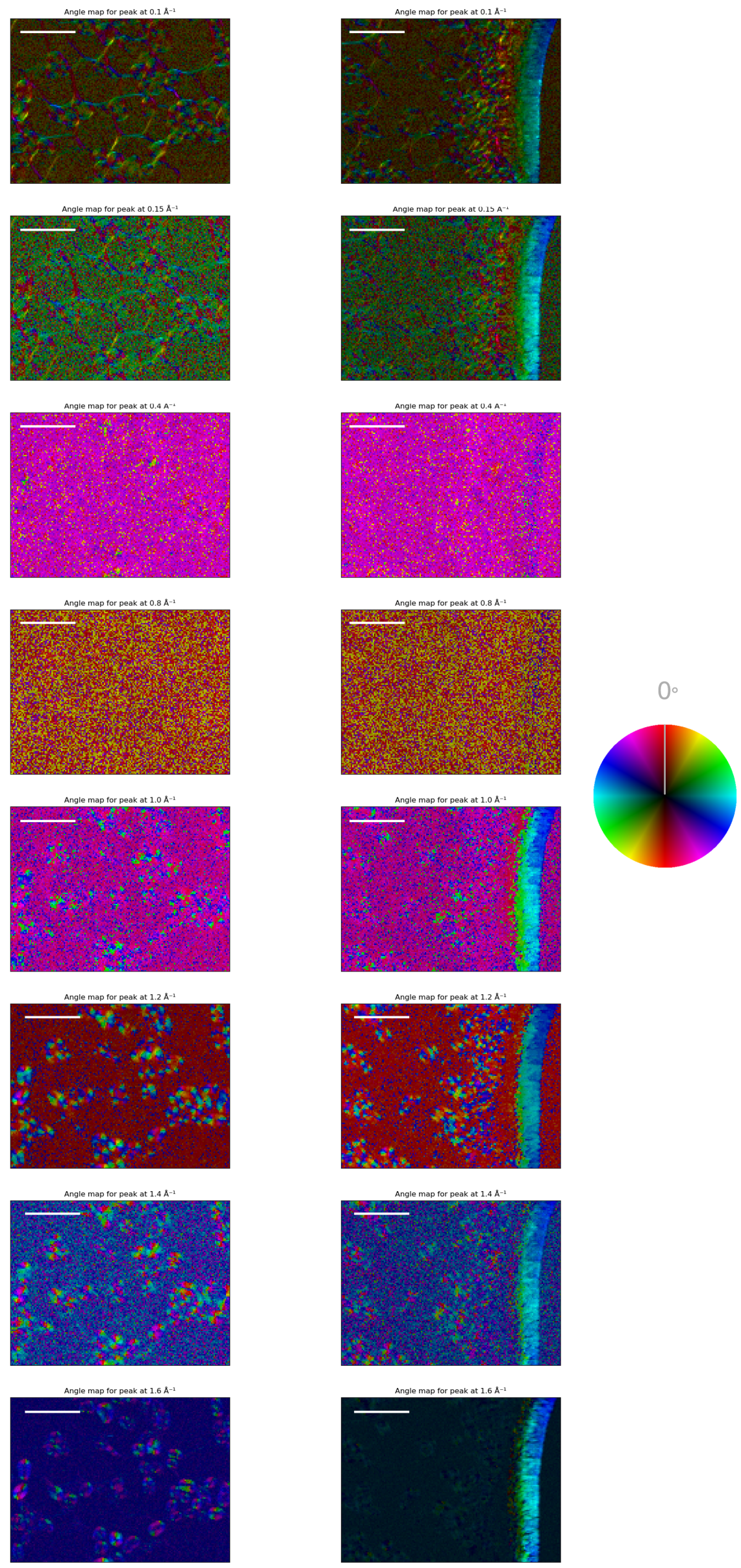

Figure S 8. Orientational angle maps based on the azimuthal profiles at various q-values. Angular directions were determined by the maximum of the denoised azimuthal profile. The q-values were chosen based on the observed features in the radial curves. The angles are mapped by the color wheel, the grey line indicates 0°. Scale bars are 100 μm.

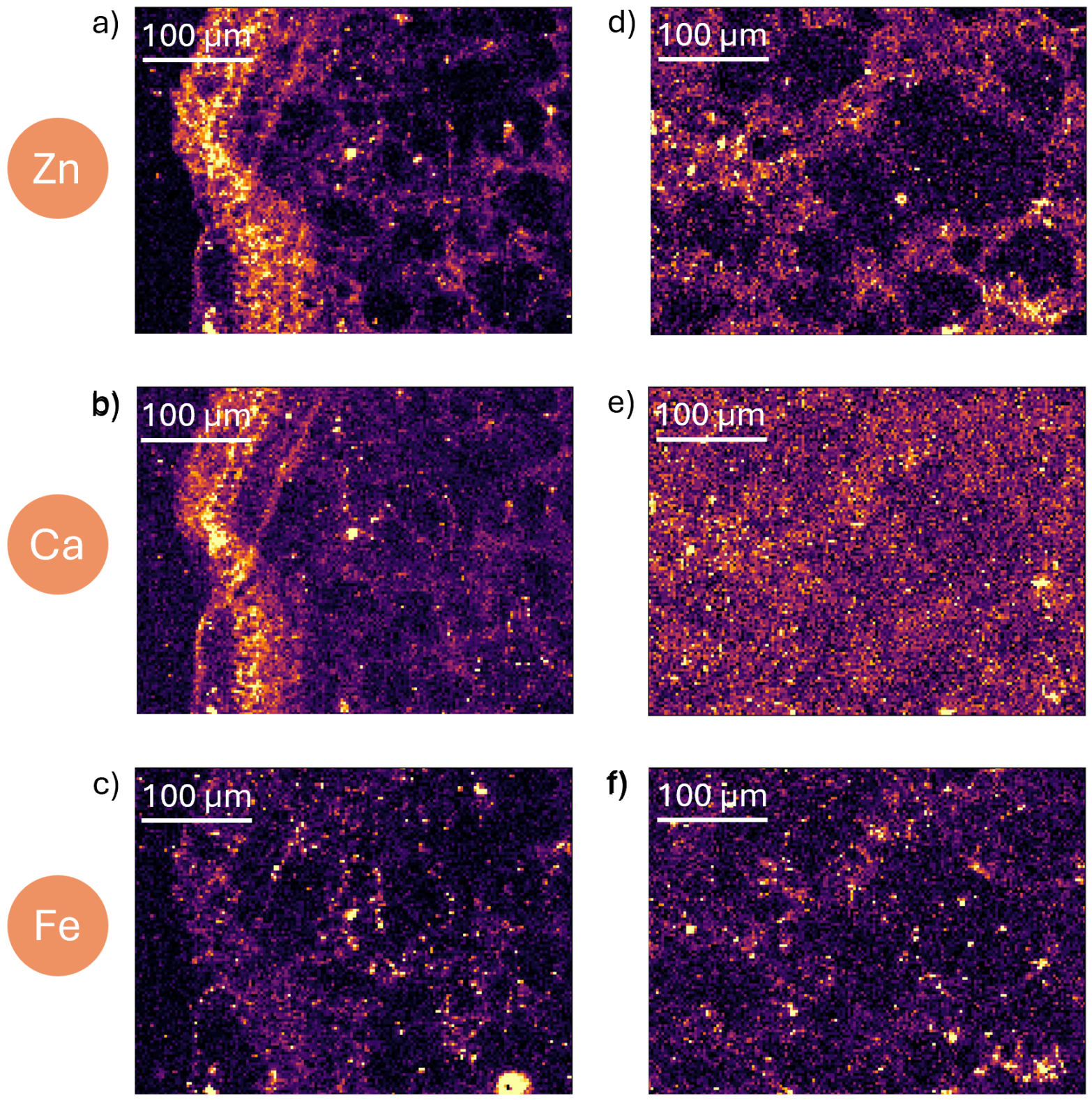

Figure S 9. X-ray fluorescence maps for more samples and the same three chemical elements as in Figure 5 in the main text. XRF maps for (a, d) zinc, (b, e) calcium and (c, f) iron.

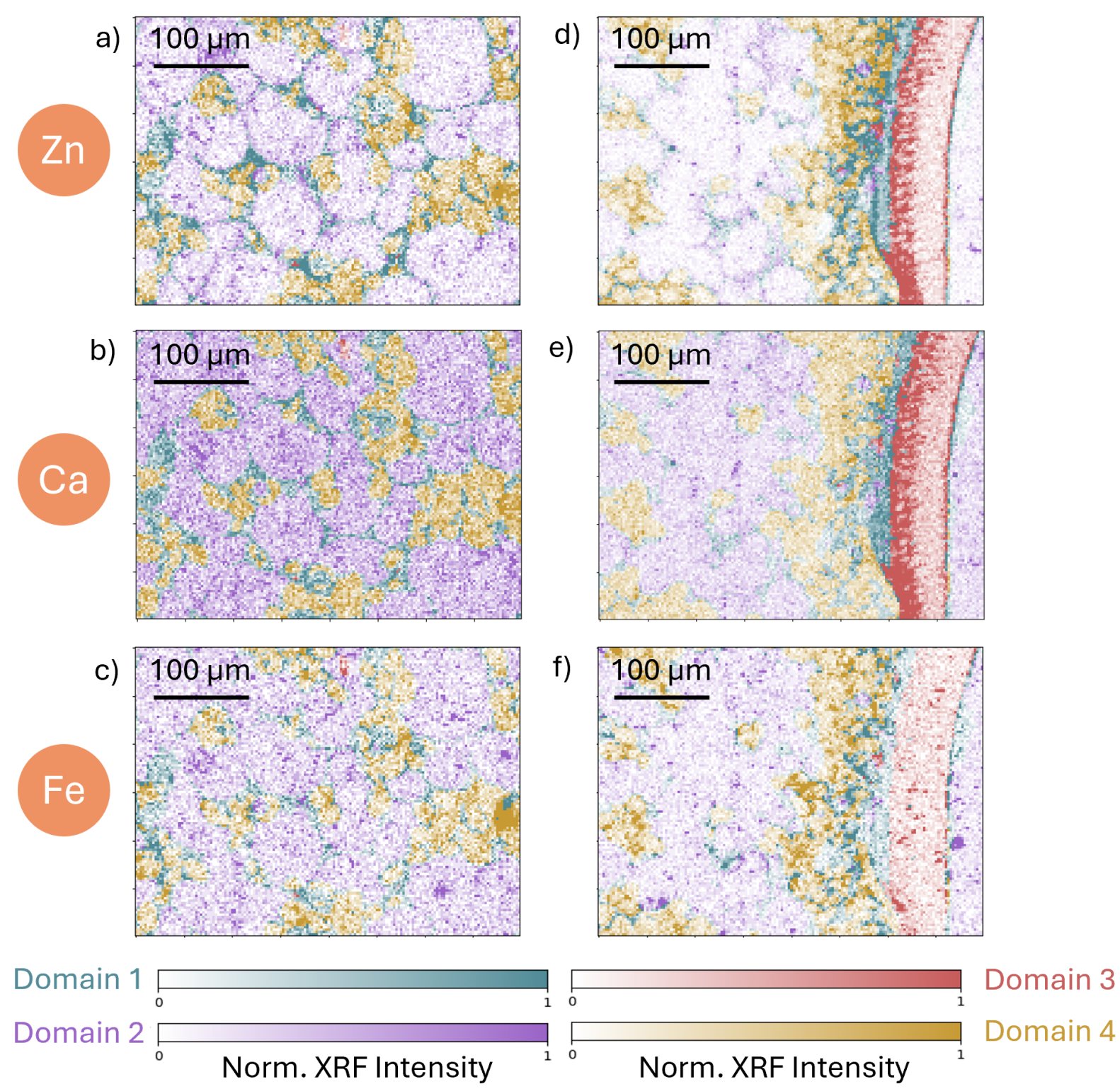

Figure S 10. Overlay of segmented maps with XRF intensity. The structural domains are identified by color hue, while opacity encodes the normalized XRF intensity. Note the adjusted colors of the domains respective to the other images, to create a colormap with comparable brightness values between the colors. (a – c) The sample from the interior of the pea, (d – f) sample containing the seed hull. (a, d) using XRF intensities for Zn, (b, e) for Ca and (c, f) for Fe. .

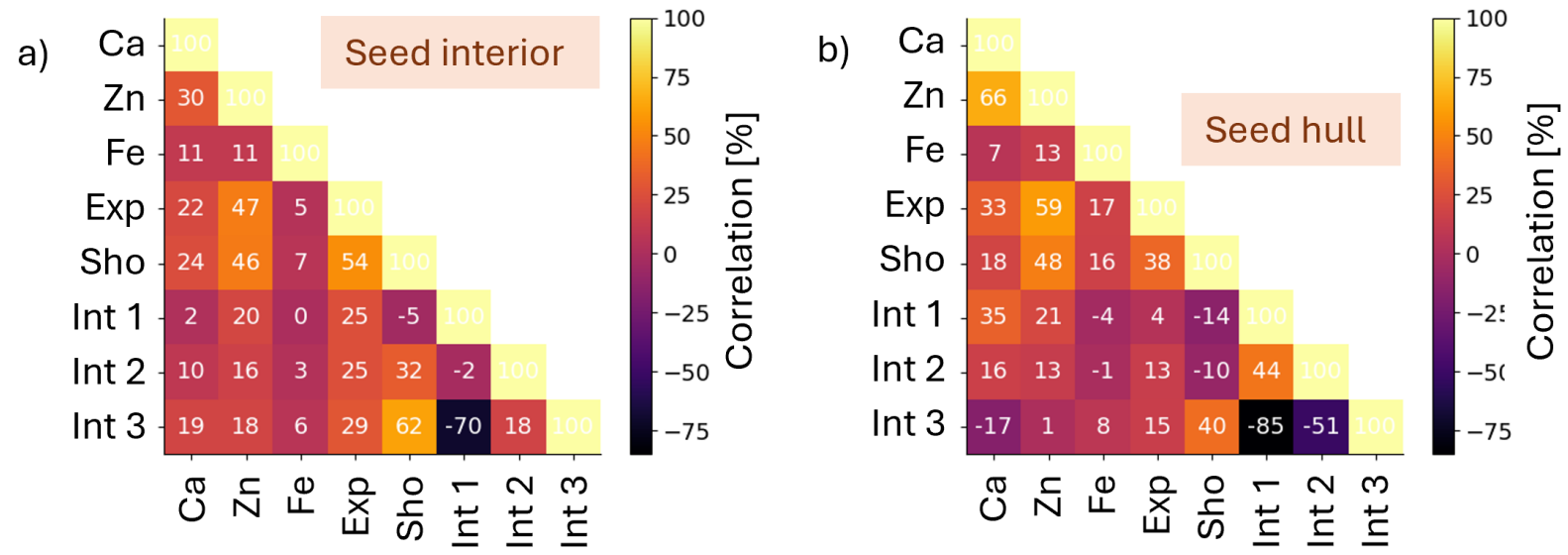

Figure S 11. Correlation matrices of selected fit parameters and XRF signals. (a) For the interior sample and (b) for the sample with seed hull. Abbreviated tick labels are: Zn, Ca, Fe – XRF signal of the respective element; Exp – power law exponent; Sho – shoulder area; Int1,2,3 – Intensities of the first, second and third Gaussian fit, as described in section 3.1 in the main text and depicted in Figure S1.

REFERENCES

[1]Christine Gernat, Sylvia Radosta, Horst Anger, and Gregor Damaschun. Crystalline parts of three different conformations detected in native and enzymatically degraded starches. *Starch - Stärke*, 45(9):309–314, January 1993.

[2]P. Cairns, T.Ya. Bogracheva, S.G. Ring, C.L. Hedley, and V.J. Morris. Determination of the polymorphic composition of smooth pea starch. *Carbohydrate Polymers*, 32(3–4):275–282, March 1997.
